# Automated Machine Learning Model Development for Intracranial Aneurysm Treatment Outcome Prediction: A Feasibility Study

**DOI:** 10.3389/fneur.2021.735142

**Published:** 2021-11-29

**Authors:** Chubin Ou, Jiahui Liu, Yi Qian, Winston Chong, Dangqi Liu, Xuying He, Xin Zhang, Chuan-Zhi Duan

**Affiliations:** ^1^Neurosurgery Center, Department of Cerebrovascular Surgery, The National Key Clinical Specialty, The Engineering Technology Research Center of Education Ministry of China on Diagnosis and Treatment of Cerebrovascular Disease, Guangdong Provincial Key Laboratory on Brain Function Repair and Regeneration, The Neurosurgery Institute of Guangdong Province, Zhujiang Hospital, Southern Medical University, Guangzhou, China; ^2^Department of Biomedical Sciences, Faculty of Medicine and Health Sciences, Macquarie University, Sydney, NSW, Australia; ^3^Monash Medical Centre, Monash University, Clayton, VIC, Australia; ^4^Department of Neurosurgery, The First People's Hospital of Foshan, Foshan, China

**Keywords:** intracranial aneurysm, AutoML, endovascular treatment, machine learning, stroke

## Abstract

**Background:** The prediction of aneurysm treatment outcomes can help to optimize the treatment strategies. Machine learning (ML) has shown positive results in many clinical areas. However, the development of such models requires expertise in ML, which is not an easy task for surgeons.

**Objectives:** The recently emerged automated machine learning (AutoML) has shown promise in making ML more accessible to non-computer experts. We aimed to evaluate the feasibility of applying AutoML to develop the ML models for treatment outcome prediction.

**Methods:** The patients with aneurysms treated by endovascular treatment were prospectively recruited from 2016 to 2020. Treatment was considered successful if angiographic complete occlusion was achieved at follow-up. A statistical prediction model was developed using multivariate logistic regression. In addition, two ML models were developed. One was developed manually and the other was developed by AutoML. Three models were compared based on their area under the precision-recall curve (AUPRC) and area under the receiver operating characteristic curve (AUROC).

**Results:** The aneurysm size, stent-assisted coiling (SAC), and posterior circulation were the three significant and independent variables associated with treatment outcome. The statistical model showed an AUPRC of 0.432 and AUROC of 0.745. The conventional manually trained ML model showed an improved AUPRC of 0.545 and AUROC of 0.781. The AutoML derived ML model showed the best performance with AUPRC of 0.632 and AUROC of 0.832, significantly better than the other two models.

**Conclusions:** This study demonstrated the feasibility of using AutoML to develop a high-quality ML model, which may outperform the statistical model and manually derived ML models. AutoML could be a useful tool that makes ML more accessible to the clinical researchers.

## Introduction

Endovascular therapy is widely used in the treatment of intracranial aneurysms (1). Despite a remarkable advancement of the endovascular coiling for intracranial aneurysms, there still exists a high rate of recurrence and recanalization. It has been reported that the recanalization rate for coiling and flow diversion are 20.8 and 10.2%, respectively (2, 3). Approximately up to 50% of patients who succumbed to recurrence or recanalization necessitated further treatment, which may place an additional financial burden on the patients. Moreover, recanalization puts patients at increased risk of a thromboembolic event or aneurysm rupture. Many studies have tried to study the risk factors for recanalization. The aneurysm size, morphologies, treatment strategies, and hemodynamics have been found to be associated recanalization (4–9). Some studies have tried to develop the models or grading scales to predict treatment outcome (4, 10–12). However, evaluation of some of the grading scales showed relatively poor performance (13).

In recent years, the machine learning (ML) models, as an alternative to the conventional statistical model, have shown promise in many clinical areas (10, 14, 15). ML models can learn complex relationships from a large amount of data. Compared with a regression model that focus on statistically significant variables, the ML models can discover non-intuitive patterns from variables which may be overlooked by statistical test (16).

Although the ML models have shown outstanding performance, the development of such models requires expertise in ML. Despite the existence of open-source code libraries, such as Scikit-Learn, PyTorch, and Tensorflow, their use still requires significant experience in programming and knowledge of ML. In addition, a high-quality model usually requires expertise to tune and train. All these problems pose a great challenge for the clinical researchers hoping to adopt ML in their research.

The recently emerged automated machine learning (AutoML) has found a way to close the gap between ML and non-artificial intelligence (non-AI) experts. The emergence of AutoML automates the process of building an ML model which in the past relied on data scientists. This lowers the learning threshold for using ML and allows people without expertise in ML to apply ML to their own area. It has recently been reported that AutoML has helped the physicians to develop the ML models that achieved good performance in the field of medical image analysis and disease risk prediction (17, 18). However, such success has not been reported in the field of stroke treatment.

Therefore, in this study, we aimed to evaluate the feasibility of using AutoML to develop the ML models for aneurysm treatment outcome prediction. Treatment was considered successful if angiographic complete occlusion was achieved at follow-up. We developed the prediction models for treatment outcome using three different methods: a statistical multivariate regression model, a manually derived ML model, and an AutoML derived ML model, and compared their performance.

## Methods

### Patient Cohorts

The patients were recruited according to the protocol of a prospective cohort (19). The primary endpoints of the cohort study are an evaluation of the safety and efficacy of interventional treatment for 6 months after surgery, with each participant completing at least 1 year of follow-up. Approval for this study was obtained from the local Institutional Review Board. The data used in the current study were anonymous and the requirement for informed consent was therefore waived. From the prospective cohort, we included the aneurysm cases treated by endovascular treatment. Dissecting aneurysms and fusiform aneurysms, aneurysms with prior treatment, or the cases with missing clinical information were excluded. A total of 395 patients were identified from our center. However, due to loss to follow-up or incomplete record, only 182 patients and 218 aneurysms with complete record of angiographic follow-up were used in the current study.

### Data Acquisition

The morphological parameters were measured and calculated from three-dimensional digital subtraction angiography (DSA) images prior to treatment. The measurements were done by two independent neurosurgeons and the average of their readings were used. The clinical symptoms, such as feeling of headache, nausea, vomit, and dizziness were recorded. The blood tests were also performed for the patients prior to treatment to measure lipid level and blood clotting function. Additionally, the patient demographics, medical history, and lifestyle behaviors were recorded. Treatment related parameters, such as treatment method, number of coils stent metal coverage rage (MCR) were included. Immediate angiographic outcome after treatment and follow-up angiographic outcome were also recorded according to the Raymond–Roy Occlusion Classification scale (20). Treatment was considered successful if complete occlusion was achieved at follow-up. The average follow-up time for the coiling and stent-assisted coiling (SAC) cases is 9.4 and 14.2 months for flow diversion cases. The complete list of collected variables is shown in Table 1.

**Table 1:** Result of univariate analysis.

**Variable**	**Occluded**	**Recanalized**	***P***
	**(*N* = 194)**	**(*N* = 24)**	
Gender (Female)	127	15	0.748
Age	54.9 ± 10.9	54.4 ± 11.7	0.931
Dizzy	90	9	0.409
Headache	110	11	0.312
Nausea	159	15	0.025^*^
Vomit	161	15	0.016^*^
Alcohol	25	5	0.286
Smoking	26	4	0.661
Labor work	10	1	0.835
Lack of sleep	21	5	0.297
Height	161.7 ± 7.5	164.7 ± 7.4	0.137
Weight	59.7 ± 9.0	58.3 ± 9.6	0.586
Systole	130.6 ± 18.6	129.1 ± 20.1	0.766
Diastole	80.8 ± 10.1	79.3 ± 12.3	0.890
Glucose	5.5 ± 1.8	5.6 ± 1.6	0.455
GHb	5.8 ± 0.7	5.7 ± 0.6	0.571
WBC	7.3 ± 3.1	7.7 ± 3.3	0.378
Platelet	239.0 ± 57.9	261.6 ± 65.9	0.141
Triglyceride	1.4 ± 1.4	1.1 ± 0.6	0.144
Cholesterol	4.6 ± 0.9	4.4 ± 0.8	0.639
LDL	2.7 ± 0.8	2.9 ± 0.9	0.443
HDL	1.3 ± 0.3	1.3 ± 0.3	0.997
Fibrin	3.3 ± 0.8	3.6 ± 1.1	0.238
APTT	35.7 ± 3.5	36.9 ± 3.8	0.169
PT	12.9 ± 0.7	13.1 ± 0.9	0.771
Hcy	10.7 ± 3.9	11.1 ± 6.1	0.531
Multiple	74	10	0.738
Rupture	161	20	0.966
Hypertension	55	9	0.530
ICA	121	11	0.118
MCA	26	2	0.484
ACA and AComA	26	4	0.661
PComA	12	2	0.686
Posterior circulation	9	5	0.002^*^
Irregular shape	33	8	0.054
Aneurysm size	4.9 ± 3.2	7.8 ± 4.5	0.003^*^
Sac width	4.6 ± 3.2	7.0 ± 4.5	0.013^*^
Sac height	4.4 ± 2.9	6.9 ± 4.2	0.009^*^
Neck width	4.0 ± 1.8	4.6 ± 2.2	0.094
Vessel angle	100.1 ± 29.2	111.2 ± 36.0	0.232
Parent artery	3.1 ± 0.9	3.1 ± 0.8	0.699
Size ratio	1.7 ± 1.3	2.5 ± 1.5	0.006^*^
Aspect ratio	1.1 ± 0.6	1.5 ± 0.8	0.011^*^
Previous SAH	20	0	0.256
SAC	137	9	< .001^*^
FD	19	0	0.109
Neck metal coverage	17.5 ± 10.5	12.8 ± 5.7	0.161
Post-procedure Angiographic Occlusion	30	4	0.023^*^
mRS	0.62 ± 1.01	0.51 ± 0.77	0.116

* *indicates statistical significance, P < 0.05*.

### General Procedures of ML

**Figure 1:**
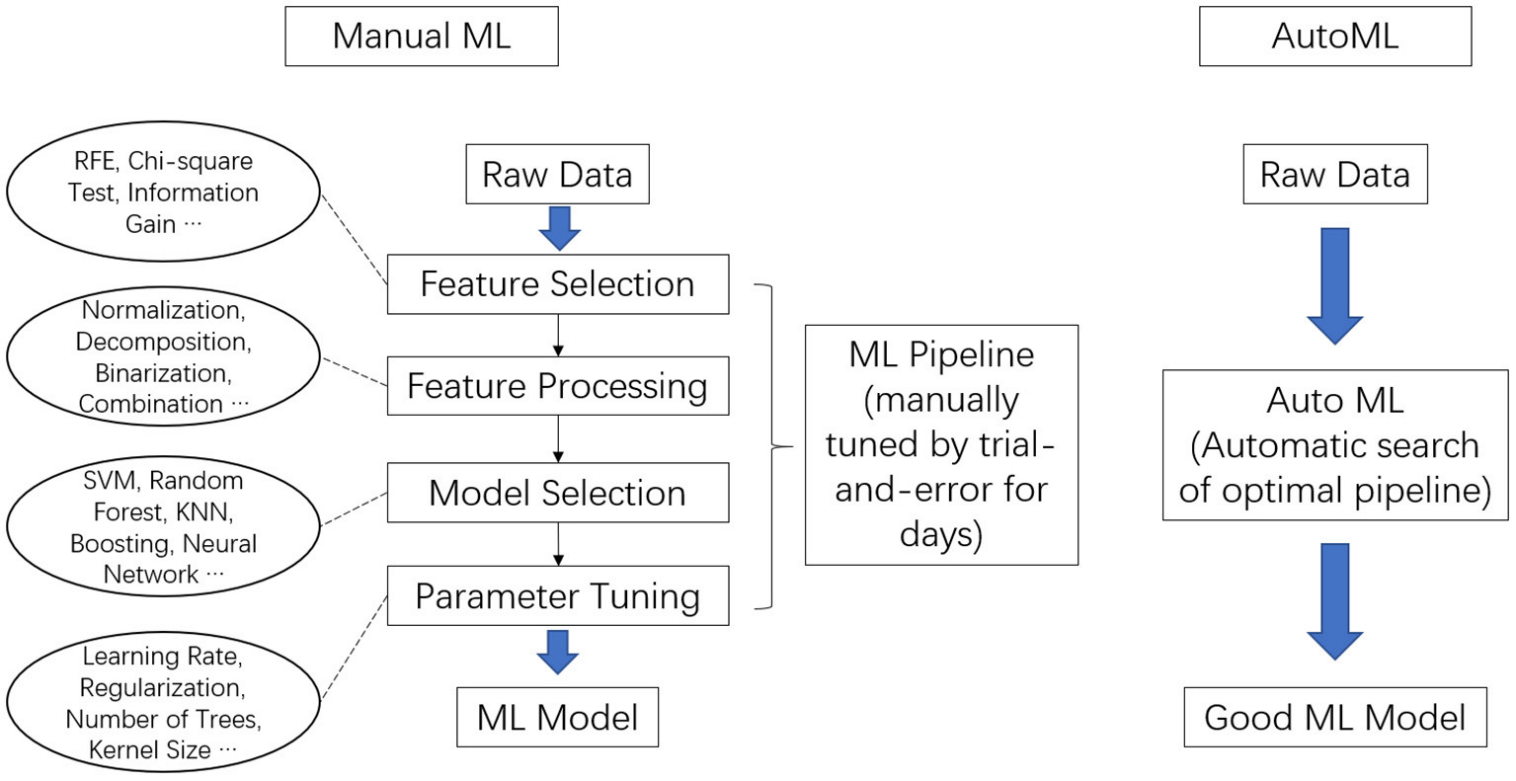
General pipeline of training a machine learning **(ML)** model (left) and training using an automated machine learning (AutoML) (right).

The general procedures of ML include the following steps: feature selection, feature engineering, ML model selection, and hyperparameter tuning, as shown in Figure 1. In feature selection, the features that are relevant to the prediction target are selected based on various criteria, such as ANOVA F-value, chi-squared statistics, univariate statistical significance *P* value, and information gain. Feature selection help to identify and focus on the useful features. In feature engineering, raw features can be normalized, binarized, decomposed, or combined to create new features, which might help to better model the data. In model selection, various ML algorithms are evaluated on the dataset and the best is selected. Common ML algorithms, to list a few, include Support Vector Machine, K-Nearest Neighbors, Decision Tree, Artificial Neural Network, Random Forest, and Naïve Bayes. All these algorithms have a wide range of hyperparameters that require careful adjustment to suit different tasks and datasets. For example, Random Forest have more than a dozen of hyperparameters, such as maximum number of tresses, maximum tree depth, maximum number of features, and minimum samples in leaf. In hyperparameters tuning, the optimal hyperparameters are usually found using grid-search or randomized grid-search over millions of possible combinations of hyperparameters.

A pipeline consists of a combination of specific methods for feature selection, feature engineering, ML algorithm, and a specific set of hyperparameters. To build a good ML model, one needs to identify an optimal pipeline that achieves best performance on the dataset.

### Automated Machine Learning

Automated machine learning automates the above pipelines and explores different choices of algorithms, feature selection and feature engineering technique, and hyperparameters. Since each major step in the pipeline involves dozens to millions of choices, complete exploration of all possible pipelines is inefficient and impractical. To speed-up the search process, we employed an algorithm called Tree-based Pipeline Optimization (TPOT) to automate the pipeline search. TPOT is based on the evolutionary algorithm which uses genetic programming to search for optimal pipeline (21). Genetic programming mimics the way of natural selection. Briefly, in each optimization run (generation), TPOT randomly generates multiple pipelines (population). These pipelines were evaluated based on their accuracy (fitness to survive). The best few pipelines (scored by accuracy) were selected into the next optimization run (selection). The selected pipelines were then randomly modified (mutation and crossover) in which a few of the pipeline elements (e.g., ML models, feature selection methods, and feature processing method) are changed. Several generations are run and the pipeline that performed best on the training set is selected as the optimal pipeline.

In the current study, AutoML was used on the training set to obtain an optimal pipeline. To avoid overfitting, 10-fold cross-validation was used. For the setting of AutoML, the number of generations to run was set to 10 and the population size at each generation was 100. Increasing the number of generations or the population size can result in higher chance of discovering better pipelines but at the cost of computational time. In the current study, the program was run on a desktop computer (CPU: Intel i7 8700) for ~1 h.

**Figure 2:**
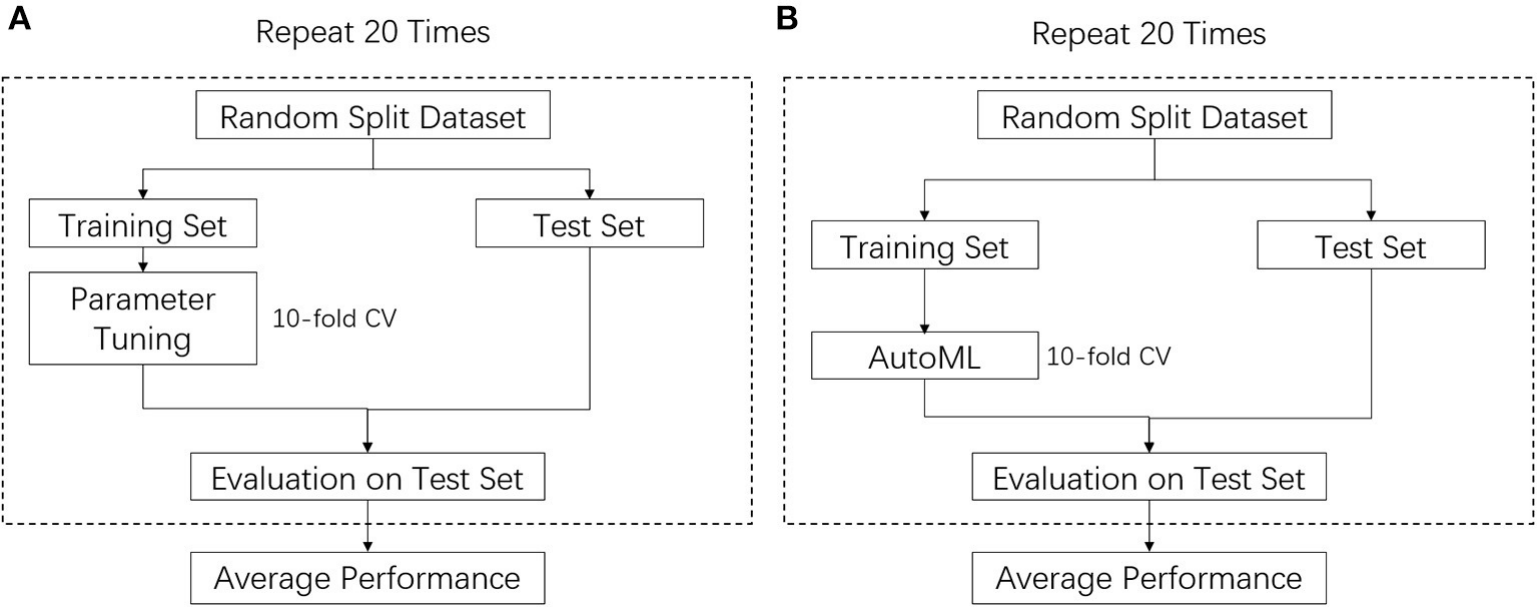
Training and evaluation procedures for manual ML **(A)** and AutoML **(B)**.

After obtaining the optimal pipeline, the derived model was evaluated on the test set. To further avoid overoptimistic results due to random split of the training and test set, the above procedures were repeated 20 times and each time with a different split of training and test set. The average performance from the 20 repeats was reported. The training and evaluation procedures are shown in Figure 2.

### Conventional ML

For comparison purposes, an ML model was trained manually using a typical method found in the literature. Random forest is one of the most popular algorithms used in the literature and is often found to have better performance than logistic regression (22–24). To represent a typical scenario in which a non-ML-expert develops an ML model for clinical research, we applied the same training procedures as described in the work of Rubber et al. The manual pipeline started with feature processing using normalization, and model building using the random forest algorithm. The hyperparameter of the algorithm (number of trees) was tuned between 5 and up to 5,000 (24). The hyperparameters were tuned using 10-fold cross-validation on the training set and the model was tested on the test set. The above procedures were repeated 20 times and each time with a different split of training and test set. The average performance of the 20 repeats was reported. The training and evaluation procedures that were used are shown in Figure 2.

### Statistical Model Building

All variables of the successful and unsuccessful cases were compared using the univariate analyses. For binary or categorical variables, the Fisher's exact test or chi-square test was performed. For continuous variables, they were first examined with the Shapiro–Wilk test to determine normality, followed by Student's *t*-test (for normally distributed variables) or Mann–Whitney *U*-test (for non-normally distributed variables). The variables with *P* < 0.05 in the univariate analysis were further selected into multivariate analysis using a backward conditional stepwise method. The statistical analyses were performed using SPSS (IBM Corporation, NY, USA). The variables that remained statistically significant (*P* < 0.05) in multivariate analysis were used for the statistical model building. For a fair comparison with other methods, a logistic regression model was fitted on the training set and evaluated on the test set. The training and evaluation procedures were also repeated 20 times and each time with a different split of training and test set. The average performance from the 20 repeats was reported.

### Aneurysm Recanalization Stratification Scales (ARSS)

For comparison with the currently used method, we chose the Aneurysm Recanalization Stratification Scales (ARSS) proposed by Ogilvy et al. (25). The scale was calculated by assigning different weights to different risk factors. Aneurysm-specific factors include size (> 10 mm), 2 points; rupture, 2 points; presence of thrombus, 2 points. Treatment-related factors include stent assistance, −1 point; flow diversion, −2 points; Raymond-Roy 2 occlusion, 1 point; Raymond-Roy 3 occlusion, 2 points. We evaluated the same test set used in the other three methods for assessing the averaged performance in 20 repeats.

### Model Comparison

Though unsuccessful cases only consist of a small portion (11%) of the dataset, it is more important to identify the unsuccessful cases than the successful cases. To avoid bias introduced by imbalanced data, besides the commonly used receiver operating characteristic (ROC) curve, we also used the precision-recall curve (area under the precision-recall curve [AUPRC]) as the evaluation metric, which is more informative than ROC when evaluating classifier on imbalanced data (26). The precision-recall curve plots precision, also termed as positive predictive value (PPV), against recall (sensitivity). The AUPRC is a balanced measure of the capability of a model to predict unsuccessful cases. The comparison of the performances of three models in the 20 repeats was examined by Wilcoxon signed ranks test as suggested by a previous study (27).

## Results

A total of 182 patients with 218 aneurysms were included. The average aneurysm size was 5.3 mm. The majority of them were located on the internal carotid artery (ICA), followed by the middle cerebral artery (MCA) and anterior communicating artery (AComA). At follow-up, only 24 cases remained unoccluded. The baselines for the successfully treated and unsuccessfully treated group are summarized in Table 1. In the univariate analysis, aneurysm size, aneurysm width, aneurysm height, presence of nausea, presence of vomit, use of SAC, aneurysm location in the posterior circulation, and the immediate post-procedure angiographic outcome showed statistical significance. In the multivariate analysis, only aneurysm size, use of SAC, and posterior circulation remained as significant variables, as shown in Table 2.

**Table 2:** Result of multivariate analysis.

**Variable**	**OR**	***P***
Aneurysm size	1.242 (95% CI 1.090-1.416)	0.001
SAC	0.208 (95% CI 0.079-0.546)	0.001
Posterior circulation	4.383 (95% CI 1.046-18.370)	0.043

**Figure 3:**
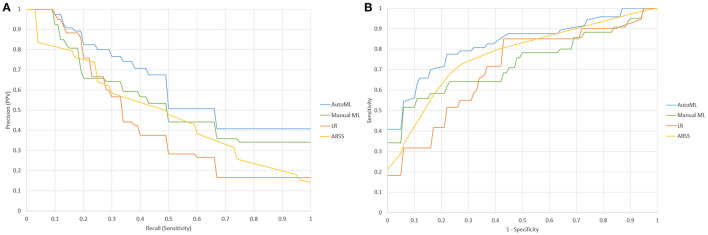
**(A)** Precision-recall characteristic curves of a statistical model **(LR)**, manually derived ML model (Manuel), AutoML derived ML model (AutoML), and Aneurysm Recanalization Stratification Scale **(ARSS)**; **(B)** the receiver operating characteristic **(ROC)** curves of statistical model **(LR)**, ManualML, AutoML, and ARSS.

The sensitivity, positive predictive value, area under the receiver operating characteristic curve (AUROC), AUPRC, and F1-score of the three models are summarized in Table 3. The statistical model achieved an AUPRC of 0.432 (95% *CI* 0.373–0.491), as shown in Figure 3. The manually derived ML model achieved better performance, with a value of 0.545 (95% *CI* 0.458–0.632). The ARSS model achieved an AUPRC of 0.496 (95% *CI* 0.418–0.574). The AutoML derived model achieved the best performance with an AUPRC of 0.632 (95% *CI* 0.585–0.679). The AUPRC of AutoML derived model was significantly higher than that from the statistical model (*P* < 0.001) and that from manual derived ML model (*P* = 0.021) and that from the ARSS model (*P* = 0.011).

**Table 3:** Summary of model performance.

	**Statistical**	**Manual ML**	**Auto ML**	**ARSS**
Sensitivity	1.000	1.000	1.000	1.000
PPV	0.167	0.342	0.408	0.142
AUROC	0.745	0.781	0.823	0.771
AUPRC	0.432	0.545	0.632	0.496
F1-score	0.286	0.508	0.578	0.378

**Figure 4:**
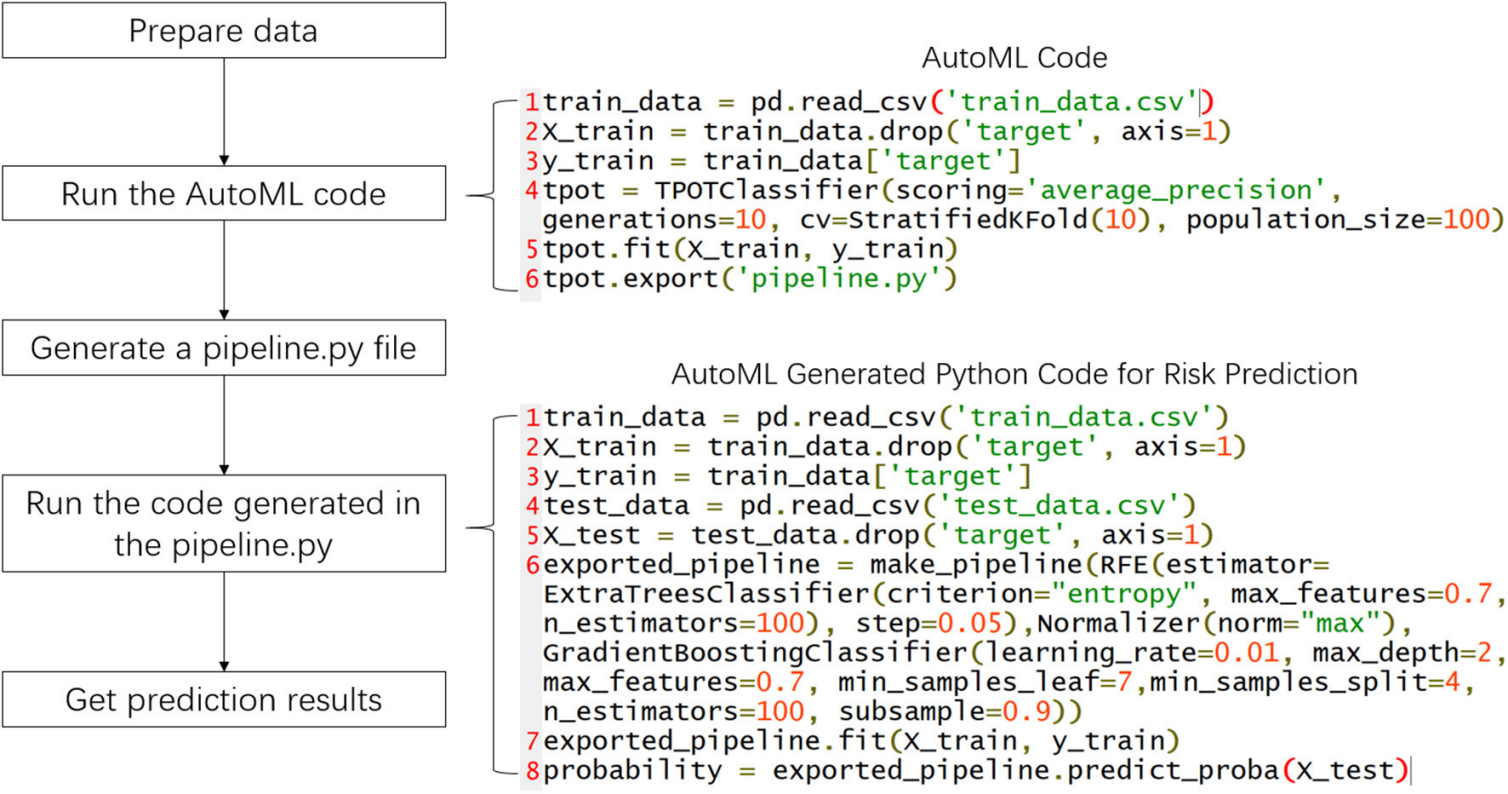
General procedures to apply AutoML in the clinical settings.

The procedures of applying AutoML in clinical settings are shown in Figure 4. The surgeons first prepare data and then run the few lines of code of AutoML and get an automatically generated Python file that contains the optimal pipeline to build a high-quality ML model. The surgeons can then use the generated python code to train an ML model and predict the risk of recanalization. In the current study, the optimal pipeline obtained started with feature selection using recursive feature elimination with Extra-Trees classifier, followed by feature preprocessing using Normalization. The algorithm used to build the model was the Gradient-Boosting classifier.

## Discussion

Recanalization and recurrence are the Achilles' Heel of endovascular treatment. This can only be confirmed by a long-term follow-up study. Thus, the question is raised: are there any methods to predict the long-term outcome of embolization? Recently, the ML models have emerged as alternatives to the traditional statistical models used to predict disease risk and therapeutic effect. However, ML is often recognized as complicated technology accessible only to a small fraction of medical researchers and data scientists. The advantage of AutoML is that it allows non-ML experts to utilize the ML models without prior expertise. In this study, we found that AutoML, with the only minimum amount of code, could develop an ML model that performed significantly better than the commonly used statistical model in predicting treatment success.

### Comparison of the AutoML Model and Statistical Model

While the statistical models are easy to derive and understand, they have several limitations. They assume linear independence between the variables which may fail to account for interactions between the variables. The prescreening of variables using *P* values may also miss important variables which may not appear statistically significant in a univariate test (28). In contrast, the ML models can learn nonlinear and interactive patterns between variables and thus producing a more accurate prediction model. Many studies have reported that an ML model outperformed the statistical models (22–24). However, there are several drawbacks that limit the use of the ML model in clinical research. One is the black-box problem of an ML algorithm yet this can be improved by applying model interpretation techniques, such as SHAP (29) to explain the prediction made by the ML models. The other problem is that the development of the ML model requires expertise in ML and usually requires the time-consuming tuning of dozens of parameters. We have shown in the current study that this can be improved by using the recently emerging AutoML technique. AutoML can make ML model training more accessible to non-ML experts without compromise in model performance.

### Comparison of an AutoML Model and Manually Derived ML Model

We have demonstrated that an AutoML derived model can achieve better performance than a manually derived ML model. The ML models need a careful selection of algorithms and tuning of hyperparameters to achieve their best performance. However, in many clinical studies that apply ML, such tuning is usually not carried out. Therefore, the developed model may not fully exploit the power of ML. In this study, we followed the same procedures mentioned in the literature to manually develop an ML model. This represented a typical scenario in which a non-ML-expert used an open-source library to train an ML model. As a result, the manually developed model is not optimal. In contrast, AutoML can perform extensive searching of different pipelines and tuning of hyperparameters, which resulted in a better model. It has been reported that AutoML outperformed a conventional ML model manually developed by a researcher with a master's degree in computer science. Moreover, AutoML only took less than an hour to train but achieved similar or even better performance than a manually derived ML model which took days to tune (17). Another distinctive advantage of AutoML compared with the conventional ML procedure is that it is much easier to use for surgeons with limited background in ML. As we have shown, the use of the AutoML model requires only a few lines of code, which makes it more accessible to clinical doctors.

### Limitations

In the current study, all the cases were from a single center and the number of cases was relatively small. Nevertheless, we have demonstrated that the use of AutoML can help clinical researchers develop high quality ML models that outperformed the statistical models and manually trained ML models. Though the current study is a single-center study with limited cases and follow-up time, the AutoML method presented in the current study can be easily generalized to a study with a larger sample size and longer follow-up time. In the current study, the treatment strategies, such as clipping, liquid embolization, or flow disruption were not assessed. To further test the applicability of our model, more cases from multiple centers with longer follow-up should be analyzed.

## Conclusions

We have demonstrated the feasibility of using AutoML to develop high quality ML model for aneurysm treatment outcome prediction. The AutoML derived model accurately predicted the outcome of treatment, which may facilitate treatment planning. AutoML may outperform the conventional statistical model and manually derived machine learning model. The emergence of AutoML simplifies and automates the process of building an ML model, which lowers the learning threshold of ML and allows non-AI experts to apply ML to their research.

## Data Availability Statement

The original contributions presented in the study are included in the article/Supplementary Material, further inquiries can be directed to the corresponding author/s.

## Ethics Statement

The studies involving human participants were reviewed and approved by Zhujiang Hospital of Southern Medical University. The Ethics Committee waived the requirement of written informed consent for participation.

## Author Contributions

CO completed the code, result analysis, and manuscript draft. JL completed the data preprocessing and result analysis. YQ, WC, and DL edited the manuscript. JL, XZ, and XH collected the data. XZ and C-ZD supervised the study and edited the manuscript. All authors contributed to the article and approved the submitted version.

## Funding

This study was supported by the National Natural Science Foundation (Grants 81974177 and 81974178) and National Key Research and Development Program (Grants 2016YFC1300804 and 2016YFC1300800).

## Conflict of Interest

The authors declare that the research was conducted in the absence of any commercial or financial relationships that could be construed as a potential conflict of interest.

## Publisher's Note

All claims expressed in this article are solely those of the authors and do not necessarily represent those of their affiliated organizations, or those of the publisher, the editors and the reviewers. Any product that may be evaluated in this article, or claim that may be made by its manufacturer, is not guaranteed or endorsed by the publisher.

## Abbreviations

AutoML: automatic machine learning
ML: machine learning
AUPRC: area under precision-recall curve
AUROC: area under receiver-operating characteristic curve
SAC: stent-assisted coiling
FD: flow diversion.
