# Supplementary material for: Automated Machine Learning Model Development for Intracranial Aneurysm Treatment Outcome Prediction: A Feasibility Study
Source: Front Neurol. 2021 Nov 29;12:735142. doi: 10.3389/fneur.2021.735142 (PMC8666475; doi:10.3389/fneur.2021.735142)
Supplement: Supplementary file 1 [file Data_Sheet_1.docx]

**Supplemental Materials**

Supplemental Table 1. Pipelines explored by TPOT

| Category | Method |
| --- | --- |
| Classifier Algorithms | GaussianNB, BernoulliNB, MultinomialNB, ExtraTreesClassifier, RandomForestClassifier, GradientBoostingClassifier, KNeighborsClassifier, LinearSVC, LogisticRegression, XGBClassifier, SGDClassifier, MLPClassifier |
| Feature Preprocessing Techniques | Binarizer, FastICA, FeatureAgglomeration, MaxAbsScaler, MinMaxScaler, Normalizer, Nystroem, PCA, PolynomialFeatures, RBFSampler, RobustScaler, StandardScaler, ZeroCount, OneHotEncoder |
| Feature Selection Methods | SelectFwe, SelectPercentile, VarianceThreshold, Recursive Feature Elimination (RFE) |

Supplemental Table 2. Performance of other popular machine learning algorithms

|  | AUROC (95% CI) | AUPRC (95% CI) |
| --- | --- | --- |
| SVM | 0.753 (0.693-0.813) | 0.489 (0.395-0.582) |
| Gaussian Naive Bayes | 0.713 (0.616-0.810) | 0.453 (0.350-0.556) |
| Multilayer Perceptron | 0.613 (0.503-0.723) | 0.461 (0.338-0.583) |
